# Arthropod Ectoparasites of Two Rodent Species Occurring in Varied Elevations on Tanzania’s Second Highest Mountain

**DOI:** 10.3390/biology12030394

**Published:** 2023-03-02

**Authors:** Genet B. Gebrezgiher, Rhodes H. Makundi, Abdul A. S. Katakweba, Steven R. Belmain, Charles M. Lyimo, Yonas Meheretu

**Affiliations:** 1 African Centre of Excellence for Innovative Rodent Pest Management and Biosensor Technology Development, Sokoine University of Agriculture, Morogoro P.O. Box 3110, Tanzania; 2 Institute of Pest Management, Sokoine University of Agriculture, Morogoro P.O. Box 3110, Tanzania; 3 Department of Wildlife Management, Sokoine University of Agriculture, Morogoro P.O. Box 3073, Tanzania; 4 Department of Biology, Mekelle University, Mekelle P.O. Box 231, Ethiopia; 5 Natural Resources Institute, University of Greenwich, Chatham Maritime ME4 4TB, UK; 6 Department of Animal, Aquaculture and Range Sciences, Sokoine University of Agriculture, Morogoro P.O. Box 3004, Tanzania; 7 Institute of Mountain Research and Development, Mekelle University, Mekelle P.O. Box 3102, Ethiopia; 8 Department of Wildlife, Fish and Environmental Studies, Swedish University of Agricultural Sciences, 901 83 Umea, Sweden

**Keywords:** *Montemys delectorum*, *Rhabdomys dilectus*, *Varroa* mite, PCR, fleas, Mount Meru

## Abstract

**Simple Summary:**

The interaction of small mammals in the ecosystem is not limited to humans and other wildlife; it also includes organisms that inhibit their bodies, so-called “parasites”. Arthropod ectoparasites are a diverse and well-adapted group of invertebrates that live on the body surfaces of their hosts, typically vertebrates but rarely other invertebrates. Ectoparasites such as fleas and some mite species are of veterinary and medical importance because they are associated with the transmission of zoonotic diseases. The study determined factors influencing ectoparasite infestation on two rodent species on Mount Meru, one of Tanzania’s most popular research and ecotourism sites. Host sex, species, and environmental temperature predicted ectoparasite infestation patterns in the two rodent species. We expected host density to predict parasite prevalences and abundances, because hosts in higher densities should have more parasites due to increased contact between individuals. However, temperature, not host density, affected ectoparasite distribution. Since temperatures decrease with elevation, parasite prevalences and abundances were lower at higher elevations, highlighting that cold conditions at higher elevations limit reproduction and development—this shows that higher elevation zones are ideal for conservation.

**Abstract:**

Climate change causes organisms, including species that act as parasite reservoirs and vectors, to shift their distribution to higher altitudes, affecting wildlife infestation patterns. We studied how ectoparasite distributions varied with altitude using two rodent species, *Montemys delectorum* and *Rhabdomys dilectus*, at different elevations (1500–3500 m). The ectoparasites infesting the two rodent species were influenced by the host sex, species, and temperature. We expected host density to predict parasite infestation patterns, because hosts in higher densities should have more parasites due to increased contact between individuals. However, temperature, not host density, affected ectoparasite distribution. Since temperatures decrease with elevation, parasite prevalences and abundances were lower at higher elevations, highlighting that the cold conditions at higher elevations limit reproduction and development—this shows that higher elevation zones are ideal for conservation. The rodents and ectoparasite species described in this study have been reported as vectors of diseases of medical and veterinary importance, necessitating precautions. Moreover, Mount Meru is a refuge for a number of endemic and threatened species on the IUCN Red List. Thus, the parasitic infection can also be an additional risk to these critical species as well as biodiversity in general. Therefore, our study lays the groundwork for future wildlife disease surveillance and biodiversity conservation management actions. The study found a previously uncharacterized mite species in the Mesostigmata group that was previously known to be a parasite of honeybees. Further investigations may shed light into the role of this mite species on Mount Meru.

## 1. Introduction

The interaction of small mammals in the ecosystem is not limited to humans and other wildlife; it also includes organisms that inhibit their bodies, so-called “parasites”. This implies that the host serves as a habitat for the parasite, providing it with food, space to live, and mating opportunities, regardless of whether they reside inside or outside the body of the host [1,2]. Arthropod ectoparasites are a diverse and well-adapted group of invertebrates, ranging from obligatory to facultative, and permanent to intermittent; they live on the body surfaces of their hosts, particularly vertebrates [2]. Some arthropod ectoparasites are also known to parasitize invertebrates. For instance, the *Varroa* spp. (Mesostigmata mite) is a known parasite of honeybees [3,4,5,6,7]. Ectoparasites can harm hosts by feeding on their tissues and causing dermatitis, and some of them are vectors of pathogenic and life-threatening diseases. As an example, plague is a flea-borne zoonosis of mammalian hosts that causes significant human mortality throughout the world, including Africa [8,9,10,11]. Parasite infections impair host fitness, because the development of antiparasitic defenses needs resources which are depleted from those needed for life-history processes [12]. Parasites slow host growth, survival, and fecundity, which have the potential to reduce host density [13]. High ectoparasite loads, for example, resulted in lower reproduction and overwinter survival in yellow-bellied marmots (*Marmota flaviventer*) in the Italian Alps [12], hare populations (*Lepus* spp.) in the Scottish mountains [14], and red grouse (*Lagopus lagopus scoticus*) in England, which may affect host fitness and make population dynamics unstable. However, the risk of parasitic infection transmission varies with the movement and dispersal behavior of the host species across a landscape, which may alter their parasite community and increase transmission to other species [15].

The distribution of parasites among host individuals is uneven, due to host- and parasite-related characteristics and environmental factors that affect host exposure and susceptibility to parasites [16]. Host-related factors include, but are not limited to, age, body size, sex, and breeding status [17]. Parasite load may vary between age groups. Adults provide greater dietary resources for parasites than juveniles [18,19], and have a prolonged period of exposure to parasites. Additionally, parasitism may be sex-biased, with males being more heavily parasitized than females, which could be due to their larger body sizes [20], as well as their greater mobility and social contact patterns [21] in males. Parasite infection is also often higher in breeding individuals than in non-breeding individuals because reproduction is linked with increased body contact, food acquisition to meet increased nutritional and energetic demands, a change in physiology, and thus increased vulnerability to parasites [22]. However, other factors, including reduced mobility of females during pregnancy and lactation to take care of juveniles, may reduce the risk of encountering ectoparasites [23].

Parasites are not only dependent on their host, but also on favorable environmental conditions for transmission and infestation. Environmental factors that have an important regulating role in the distribution and developmental success of parasites in mammals include precipitation [24], temperature [25,26], and elevation [27,28]. Parasite distributions are anticipated to shift northward and to higher altitudes, as a result of climate change [29]. In fact, rising temperatures associated with climate change are increasing parasite abundance over time [30,31,32,33]. These distribution changes may lead to new host–parasite encounters, forcing host populations to deal with parasites with which they have not co-evolved, which could eventually result in local host population extinctions [34].

Steep mountain ranges provide a natural experimental background for studying how species distribution varies across climatic gradients [35]. Altitudinal gradients reflect substantial changes in precipitation, temperature, humidity, soil, and vegetation, with extreme environmental conditions strongly influencing the physiology and survival rate of organisms [36]. These variations have been demonstrated to affect faunal distribution across different altitudes in different geographic regions worldwide [36,37,38,39,40,41]. For example, small mammals’ distribution along elevation has been extensively studied throughout the world, and often shows a hump-shaped distribution pattern in which species richness and abundance peak at the mid-level elevations where the conditions are not too extreme (i.e., hot or cold) [37,38,39]. Studies indicate that the spatial and numerical distribution of the host population determines the distribution patterns of their parasites [42]. Thus, hosts in high densities contain a greater parasite load than hosts in low densities, because of the increased contact between individuals, making contagious parasite transmission easier [42,43]. Accordingly, we hypothesized that ectoparasite distributions follow the distribution patterns of their hosts; ectoparasites should peak at the mid-elevation. Yet, the assumption regarding parasite distribution along elevational gradients is still being debated. On the other hand, the number of feather mites in birds [44] and fleas in rodents [45] has been shown to decline as elevation increases.

This study determined the distribution of arthropod ectoparasites on the East African soft-furred mouse (*Montemys delectorum*) and the mesic four-striped grass rat (*Rhabdomys dilectus*) at different elevations on Mount Meru, the centerpiece of Arusha National Park, a popular ecotourism destination in Tanzania. *Rhabdomys* has traditionally been seen as a single species, *Rhabdomys pumilio.* However, karyotype and mtDNA evidence suggests that it includes a second species, *Rhabdomys dilectus*, which is found all over Africa [46]. *M. delectorum* is the only member of the genus *Montemys* within the family Muridae. It was formerly classified in the genus *Praomys* (i.e., *Praomys delectorum*), and was recently taxonomically revised [47]. *M. delectorum* is an endemic mammal to the East African Highlands. It is a threatened species throughout its range due to habitat loss, and its IUCN conservation status is uncertain [48]. We examined which traits of the host (sex, species, density) and environmental factors (elevation, temperature) determined the prevalence and abundance of arthropod ectoparasite vectors, which is important to understand the patterns of parasitic infectious disease. We also tested the hypothesis that ectoparasite distribution follows the distribution patterns of their host. The distribution pattern of rodents and shrews on Mount Meru is greater at intermediate elevation levels [39,41]; hence, if host density predicts the occurrence of the ectoparasites, we particularly expected rodent individuals at the middle elevations to suffer a high risk of being infested. Furthermore, the two rodent species have been identified as hosts of ectoparasites, including as potential vectors and reservoirs of pathogens [49]. Given the 65% increase in zoonotic outbreaks in Africa over the past decade [50], recognizing the patterns of parasite distribution among wildlife hosts is of major importance. Our study contributes to a better understanding of the ecology of host-ectoparasite relationships along elevational gradients, which is important not only for identifying potential ectoparasite vectors, but also for designing and implementing vector-borne disease management programs for wildlife conservation and human health.

## 2. Materials and Methods

### 2.1. Study Area and Trapping Sites

**Figure 1:**
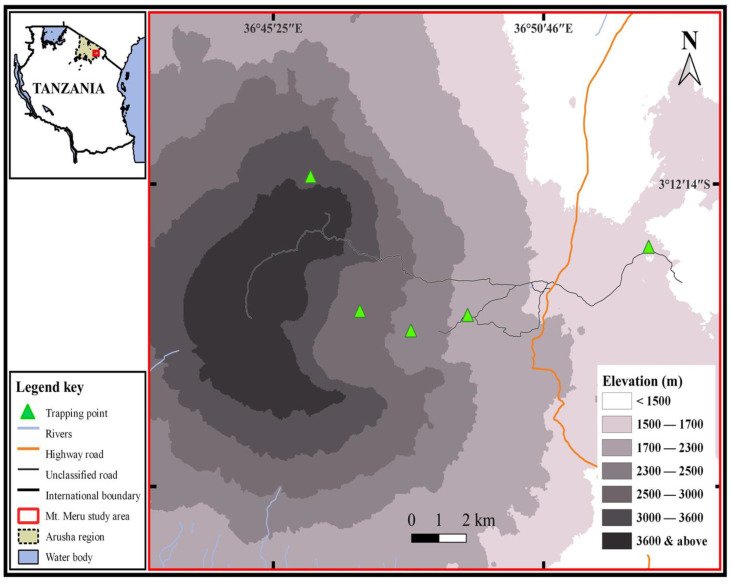
Geographic locations of trapping sites on Mount Meru.

Mount Meru is located in northeastern Tanzania at 3°14′48′′ and 36°44′54′′, about 35 km northeast of the town of Arusha (Figure 1). The topography rises from the Momela Lakes, lying at 1400 m above sea level, to the summit at 4566 m. The rainy season extends from November to May with relatively lower rain in January and February. June through October is characterized as the dry and cold season. Annual atmospheric temperatures range between 15 °C and 34 °C [51]. Details of the vegetation types of the sites from which the rodents were trapped are outlined in Gebrezgiher et al. [39] and Bussmann [52], and all trapping locations were within Arusha National Park (Figure 1). The study was conducted between February 2021 and June 2022. The sampling sites were across five elevations from 1500 to 3500 m as follows:

(1) Elevation 1500 m (3°13′20.766′′ S, 36°52′50.076′′ E)—This trapping site was located at the base of Mount Meru, and vegetation cover ranges from grassland, thicket, and bushland to woodland. *Caesalpinia decapetala*, *Croton macrostachyus*, *Jacaranda mimosifolia*, *Ocimum gratissimum*, *Solanum incanum*, *Aerva lanata*, *Lantana trifoliata*, and tussock grasses are among the notable plant species. Patches of Acacia trees are also prevalent.
(2) Elevation 2000 m (3°14′33.102″ S, 36°49′15.528″ E)—This site was situated in a lower montane forest, with a dense canopy of trees of various species, including *Diospyros abyssinica*, *Olea hochstetteri*, *Rhamnus prinoides*, and *Ficus thonningii*.
(3) Elevation 2500 m (3° 14′ 32.892′′ S, 36° 47′ 25.644′′ E)—This site was in the upper montane forest dominated by *Juniperus procera* and *Podocarpus gracilior*. Herbaceous plants, various lianas, and shrubs formed a thick understory, and the trees were often covered with epiphytes.
(4) Elevation 2950 m (3°13′28.724′′ S, 36°47′7.782″ E) —This site was characterized as a transitional zone between habitats of higher montane forest and ericaceous heath.
(5) Elevation 3500 m (3°13′6.192′′ S, 36°46′24.042′′ E)—This highest trapping site was located in the ericaceous heathland habitats dominated by Erica spp.

### 2.2. Rodent Trapping

Sherman is a foldable metal trap designed to capture live animals. Four trap lines were established at a distance of 30 to 50 m, each with 50 traps separated by 10 m. Sites received six days of trapping with a total of 200 traps. All traps were equipped with bait (peanut butter mixed with maize flour and avocado) and placed in the shade to avoid being too hot. Traps were inspected and re-baited (i.e., if the bait was found eaten by insects) every morning between 07:00 and 08:00 h. Each Sherman trap that captured an animal was replaced with a new one (i.e., previously washed). Each individual trapped rodent was first placed in a cotton bag and humanely anesthetized using diethyl ether (LOBA CHEMIEPVT. LTD.) on cotton wool, then placed in a bucket with a lid. Standard external body measurements (body, tail, hindfoot, and left ear), sex, and weight of the trapped hosts were recorded. Species identification followed Happold and Kingdon [53], and sex identification followed Hoffmann et al. [54]. The rodent species identification was confirmed using molecular (Cytochrome b) techniques. 

### 2.3. Ectoparasite Collection and Identification

The ectoparasites were combed out of the rodents, using a small shoe-like brush, into a clean, wide, and long aluminum pan. A different brush was used for each host individual to make sure ectoparasites were not spread from one to another through sharing a brush. The softer body parts of the rodents, such as the belly, ear, and tail regions, were further examined. The ectoparasites were collected with fine brushes, and each host was separately counted and preserved in a labeled Eppendorf tube (Inqaba Biotec East Africa Ltd., Nairobi, Kenya) containing 70% ethanol. Each ectoparasite was identified morphologically using a compound microscope, with the aid of available dichotomous taxonomic keys [55,56,57]. Existing procedures were employed for the microscopic examination of specimens [58,59]. The specimens were temporarily mounted in glycerol, covered with a cover slip, and viewed under a compound microscope. Moreover, to allow internal structures to be clearly visible, and for external features to be more distinct, the specimens were cleared in a 10% KOH solution, boiled for 10 min for fleas, and maintained for 24 h for mite specimens. The alkaline solution was neutralized with 10% acetic acid for 30 min. After dehydrating the specimens using a series of ethanol washes (70%, 80%, and 100%), each for 1 h, they were transferred to clove oil overnight to rehydrate prior to mounting. Dibutyl phthalate-polystyrene-xylene (DPX) was used to mount the flea specimen on a microscope slide [59]. The flea and mite species identification was further confirmed using molecular techniques.

### 2.4. Molecular Identification of Fleas and Mites

DNA was extracted from the whole body of individual fleas (16 samples) and mites (4 samples) using the Quick-DNA™ Miniprep Plus Kit (Zymo Research), according to the manufacturer’s instructions. The purity and concentration of the DNA was determined using a Nano spectrophotometer at 260 and 280 nm wavelengths. 

To identify the fleas, the cytochrome oxidase subunit II (cox2) gene was amplified using the following primer sequences: forward primer (F-Leu: TCTAATATGGGCAGATTAGTGC) and reverse primer (R-Lys: GAGACCAGTACTTGCTTTCAGTCATC) [60]. PCR amplification was performed using AccuPower^®^ PCR PreMix from Bioneer (Bioneer Corporation, Daejeon, Republic of Korea). The PCR reaction mixture for the fleas consisted of 2 μL of template DNA, 0.5 μL of forward primer, 0.5 μL of reverse primer, and 17 μL of nuclease free water in a micro-tube containing AccuPower^®^ PCR PreMix concentrate, making a total reaction volume of 20 μL. Cycling conditions consisted of an initial denaturation at 95 °C for 5 min followed by 40 cycles of 94 °C for 40 s, 56 °C for 45 s, and 72 °C for 45 s. A final extension at 72 °C for 5 min was performed to complete the extension.

For the mites, the cytochrome oxidase subunit I (cox1) gene was amplified using primer sequences as follows: forward primer (cox1-F: GTTTTGGGATATCTCTCATAC) and reverse primer (cox1-R: GAGCAACAACATAATAAGTAT) [61]. A total of 20 μL of PCR reaction mixture consisted of 2 μL of extracted DNA, 1 μL of forward primer, 1 μL of reverse primer, and 16μL of nuclease-free water in a micro-tube containing AccuPower^®^ PCR PreMix concentrate. Cycling conditions consisted of initial denaturation at 95 °C for 5 min followed by 40 cycles of 95 °C for 40 s, 47 °C for 40 s, and 72 °C for 30 s. A final extension at 72 °C for 5 min was performed to complete the extension. 

Once the PCR reaction was carried out, a 1.5% agarose gel was prepared by dissolving 1.5 g of agarose into 100 mL of sodium borate buffer and heated until the agarose had dissolved completely, and was stained with 4 μL of EZ-Vision^®^ In-Gel Solution. A volume of 4 μL of each sample was loaded into each well of the gel, and 4 μL of DNA ladder was loaded into the first well in order to indicate the size of any fragments. The voltage was set to 100 V, and electrophoresis was allowed to run for 40 min. The image of the DNA fragments was captured using Bio-Rad’s Gel Doc™ EZ Imaging System. Nine amplicons, five for fleas (2–3 per species) and four for mites, were sequenced at Macrogen Europe (Amsterdam, The Netherlands). The raw sequence data were cleaned, edited, and assembled using Geneious Prime version 2022.1.1 software [62] to obtain consensus sequences. The obtained nucleotide sequences were aligned with other ectoparasite reference sequences available in the GenBank database. A maximum likelihood phylogenetic tree was constructed with the robustness of 1000 bootstraps, using the T92/+G+1 substitution model with an AICc value of 5008.13 implemented in MEGA 11 [63]. Four nucleotide sequences for mites (accession number: OP776142, OP798020, OP798021, and OP798022) and five for fleas (accession number: OP857545, OP857546, OP857547, OP857548, and OP857549) were submitted to the GenBank.

### 2.5. Data Analysis

Quantitative descriptors of the ectoparasite species on each host species were calculated in accordance with [64]. P—Prevalence (proportion of infested host individuals) was estimated using the formula (P = Re/Rt * 100%), where Re = number of individual rodent species infested with one or more ectoparasite species and Rt = total number of examined hosts. D—Ecological index of dominance was estimated using the formula (D = Es/Et * 100), where Es = number of ectoparasites of a given species collected from the rodents and Et = total number of ectoparasite species collected from the rodents; it refers the degree to which a species is more numerous than other species in an ecological community. MA—Mean Abundance of the ectoparasites was estimated using the formula (MA = Ea/Ht), where Ea = number of ectoparasites of a given species collected from the rodent species, and Ht = total number of hosts examined for that particular parasite species. Statistics are presented as mean ± SE with bias corrections.

We employed multiple regression models to determine the effects of the independent variables (temperature, elevation, host sex, host species, and host density) on the response variables, ectoparasite infestation (prevalence and abundance). Because of the small sample size, which resulted in zero ectoparasite or low rodent host counts at some of the sites, analysis was made of the combined ectoparasites (fleas and mites) and host species. As a result, no species-specific ectoparasite or rodent models were fitted. To avoid bias due to the smaller sample size, we excluded the data of the 1500 m site (N = 6 hosts) from the regression model. Host density was described as the number of captures per trap and per night, as described in Stanko et al. [65]. The distribution of ectoparasite abundance is usually patchy, with many hosts having low parasitic loads and only a few having high parasite loads, resulting in an excess zero in the data [66]. Thus, to account for dispersion and bias due to excess zero, we employed zero-inflated negative binomial (ZINB) regression models with a log link function to assess the effects on mean ectoparasite abundance. Since 41.2% of the host population had no ectoparasites present, we, therefore, used generalized linear models with a binomial distribution (infested or not infested) linked to a logit function. A host infested at least by one ectoparasite was represented by “1,” and the host not infested was represented by “0.” The probability of being infested was referred to as the prevalence. Since elevation negatively correlates with temperature, we employed them in different models to avoid confounding effects. The best-fitting model among candidate models was selected on the basis of Akaike’s information criterion corrected for small sample sizes (AICc) [67]. The model with a lower ∆AICc value was selected as a best-fit model. The summary of parameter estimates for the fitting model is presented as estimates, SE, and 95% confidence intervals; the confidence interval which includes zero is not significant. The “lme4” and “pscl” packages in R ver. 4.2.2 [68] were used for binomial and ZINB models, respectively.

## 3. Results

### 3.1. Morphological and Molecular Identification Results of Fleas and Mites

**Figure 2:**
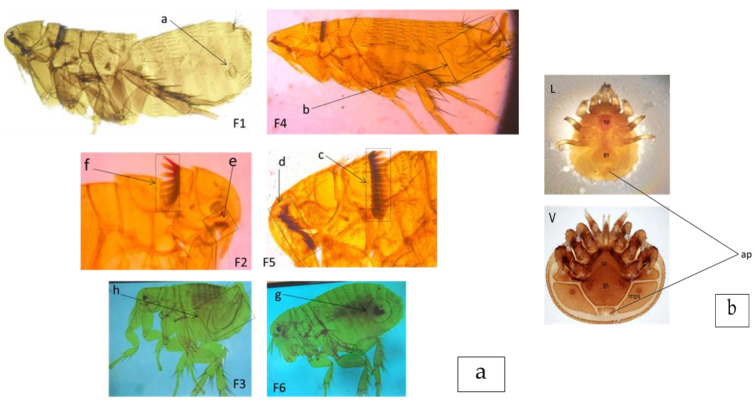
(**a**) Mounted vouchers of flea species: F1—*Dinopsyllus ellobius* (female, a—spermatheica); F2—*Ctenophthalmus calceatus cabirus* (front part; e—genal ctenidia with three spines, f—Pronotal ctenidia); F3—*Xenopsylla cheopis* (male; h—clasper); F4—*Dinopsyllus ellobius* (male, b—clasper); F5—*Dinopsyllus ellobius* (front part; c—Pronotal ctenidia, d—genal ctenidia); F6— *Xenopsylla cheopis* (male; h—clasper). (**b**) Ventral view of *Laelaps* mite (L) and *Varroa* mite (V) [69]. *Varroa* (V): Sternal plate (sp) fused with genital shield (gs); genital shield is large with lateral deep and angular projections; metapodal shields (mps) greatly enlarged and broadly triangular. *Laelaps* (L): Genital shield is flask-shaped and distinctive from the sternal plate. Both mites possess one post anal setae in the anal plate (ap).

**Figure 3:**
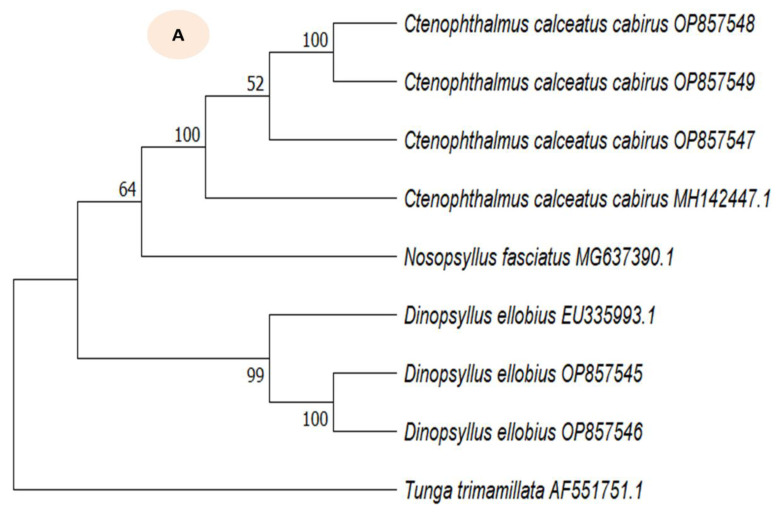

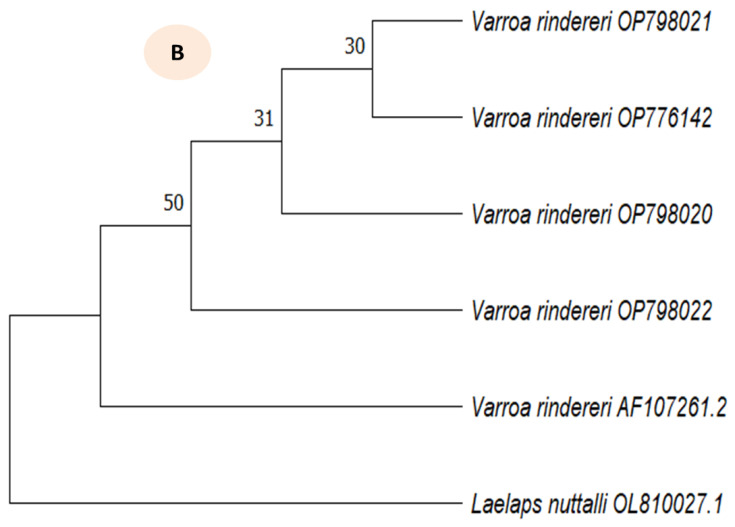
Phylogenetic tree for fleas (**A**) and mites (**B**) using cox2 and cox1 genes, respectively. The evolutionary history was inferred using the maximum likelihood method with bootstrap tests (1000 replicates). The percentage of trees in which the associated taxa clustered together is shown next to the branches. The phylogenetic tree was constructed using the sequences from this study, as well as other reference sequences from GenBank. *Tunga* and *Laelaps* were used as outgroup species for fleas and mites, respectively.

Samples of mounted vouchers of flea and mite species are provided in Figure 2a,b. BLAST search results revealed three ectoparasite species: *Ctenophthalmus calceatus cabirus* (n = 3), *Dinopsyllus ellobius* (n = 2), and *Varroa rindereri* (n = 4). The comparison *of C. calceatus cabirus* in our study with sequences obtained from *Lemniscomys striatus* from Rwanda (MH142447.1) revealed 95.19–97.89% identity of similarity in the BLAST. The phylogenetic tree for fleas (Figure 3A) was rooted using *Tunga trimamillata* as the outgroup species. *C. calceatus cabirus* (OP857547, OP857548 and OP857549) of this study shared different lineages with the reference (MH142447.1) obtained from GenBank. The percent BLAST similarity between *D. ellobius* (OP857545 and OP857546) sequences in our study and the closest match in the GenBank (EU335993.1) was 95.75%. The two *D. ellobius* sequences were clustered together, but were distantly grouped with *D. ellobius* from the references (EU335993.1). The flea, *Xenopsylla cheopis*, was not sequenced because there were not enough samples for molecular analysis; it was identified only morphologically. On the other hand, the mite species identification result in this study was ambiguous; it was identified morphologically as a *Laelaps* species (Mesostigmata). However, the cox1 gene sequence results of all four mite samples (OP776142, OP798020, OP798021, and OP798022) provided the highest-scoring BLAST hit to a sequence from the species *V. rindereri* (Order Mesostigmata) in the GenBank (AF107261.2), with an 83.24% identity of similarity. Morphologically, *Laelaps* possess a flask-shaped ventral genital shield that is distinct from the sternal plate, whereas in *Varroa* the genital shield is large, with deep lateral and angular projections that are fused with the sternal plate. Metapodal shields are greatly enlarged and broadly triangular in *Varroa* but small and inconspicuous in *Laelaps* (Figure 2b). The phylogenetic tree for mites (Figure 3B) was rooted using *Laelaps* as the out-group. The *V. rindereri* sequences in this study share different lineages with *V. rindereri* from the reference sequences (AF107261.2).

### 3.2. Quantitative Descriptors of Ectoparasite Infestation 

**Figure 4:**
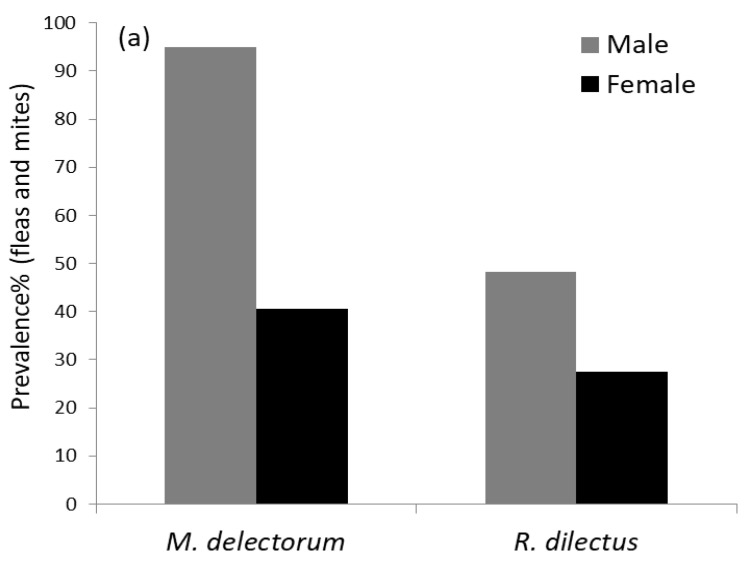

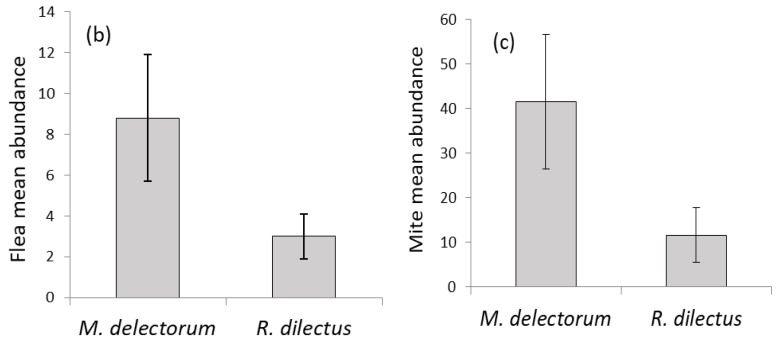
Ectoparasite infestation on *M. delectorum* and *R. dilectus*: (**a**) prevalence (%) between host sexes; (**b**) flea mean abundance with SE bar; (**c**) mite mean abundance with SE bar.

**Figure 5:**
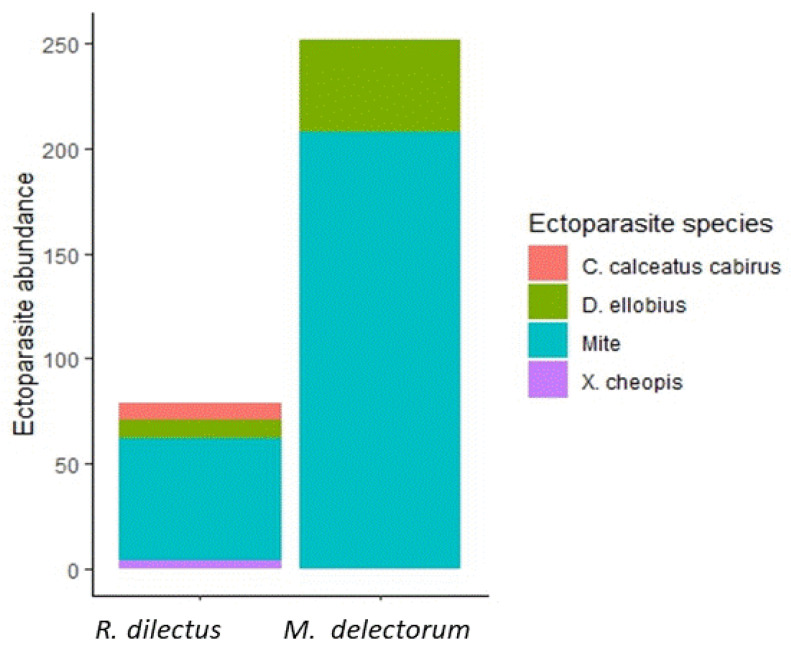
Relative contributions of flea and mite species infesting *M. delectorum* and *R. dilectus* on Mount Meru, Tanzania.

**Figure 6:**
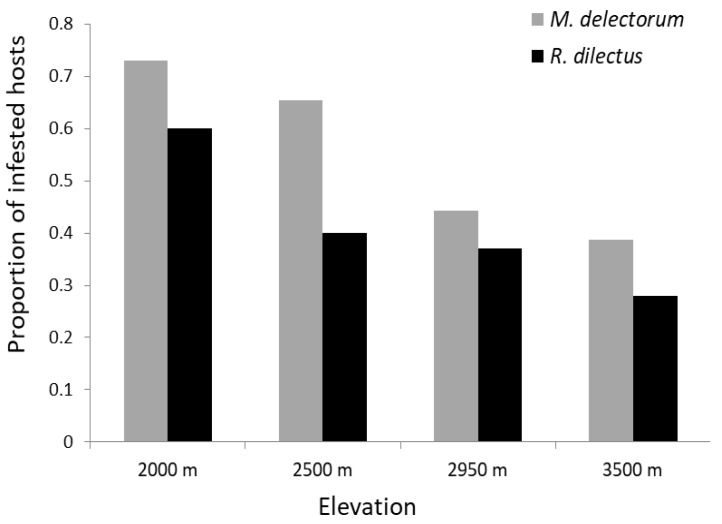
Ectoparasite prevalence of *M. delectorum* and *R. dilectus* in relation to elevation on Mount Meru.

A total of 398 rodents (335 of *Montemys delectorum* and 63 of *Rhabdomys dilectus*) were examined for flea and mite infestation (Table 1). Overall, a total of 266 mites and 59 flea individuals were recovered from the two host rodents. About 58.8% of the hosts (234/398) were infested by at least one ectoparasite. Probabilities of ectoparasite infestation of 63% and 37% were observed in *M. delectorum* and *R. dilectus*, respectively. In *M. delectorum*, 95% males and 41% females were infested, whereas in *R. dilectus*, 48.3% males and 30% females were parasitized by at least one ectoparasite (Figure 4a). The mites were recorded most frequently, accounting for 81.85% of the total ectoparasite ecological index of dominance, and contributing to 50.68% of the infestation in the total host population. Of the fleas, *D. ellobius* was the most abundant flea species, accounting for 7.11% of the total ectoparasite dominance index (Table 2). On *M. delectorum*, there were 8.8 ± 5.01 fleas and 41.6 ± 21.66 mites, whereas on *R. dilectus*, there were 3 ± 1.5 fleas and 11.6 ± 7.17 mites (Figure 4b,c). *R. dilectus* was parasitized by all the flea and mite species, whereas *M. delectorum* was infested by *D. ellobius* and mites only. In both rodent species, the mite and *D. ellobius* contributed the most to the overall parasite infestation (Figure 5). The prevalence of ectoparasites declined with increasing elevation for both host species, with the lowest record at 3500 m (Figure 6). However, the low prevalence at 1500 m (Table 1) was due to the smaller sample size of rodent hosts examined for *M. delectorum* (n = 2) and *R. dilectus* (n = 4). The flea *X. cheopis* was recorded only at 1500 m on *R. dilectus*, whereas the other ectoparasite species were found at multiple elevations (Table 1).

### 3.3. Assessment of Parameters Influencing Ectoparasite Occurrence

The results of the study show that host species, sex, and temperature best predict ectoparasite prevalence and mean abundance (Table 3, Table S1 and Table S2). M. delectorum had a higher prevalence (1.22 ± 0.30) than R. dilectus, and male individuals had a greater prevalence (2.56 ± 0.28) than female individuals. Similarly, the parasites’ mean abundance was greater in M. delectorum (0.42 ± 0.14) and in males (0.93 ± 0.12). 

## 4. Discussion

This study investigated the distribution of arthropod ectoparasites on *Rhabdomys dilectus* (mesic four-striped grass rat) and *Montemys delectorum* (East African soft-furred mouse), which are the two most abundant species, with a wide range of distribution on Mount Meru [39,41]. Mount Meru has experienced habitat disturbances due to a fire outbreak in the ericaceous habitats; deforestation; road construction along the National Park; and ecotourism activities including clearing of land for vehicle parking and camping. Moreover, the degradation of habitats by human activities, including farming and grazing, has intensified in the last decades, particularly at the base of the mountain [51]. The habitat degradation and temperature increases due to climate change have been reported to cause an upward shift in the distribution of rodent and shrew species, including endemic and threatened species on the IUCN Red List [39]. However, parasites can also be a danger to threatened species because many threatened mammal populations are fragmented and small, and have low levels of genetic diversity [70,71]. These conditions can enhance host susceptibility and exposure to parasitic infections [72]; moreover, parasitic infections could be another factor that contributes to an increased probability of stochastic extinction [73,74]. Therefore, the parasite burden observed in this study, especially for the endemic *M. delectorum* (with 63% parasite prevalence), needs attention and improved conservation management.

Rodents on Mount Meru were infested more with mites than fleas, consistent with previous studies elsewhere [75,76]. In contrast, despite the fact that fleas are the most dominant group of ectoparasites for small mammals [77], we collected far fewer fleas than mites. The most likely explanation is that fleas spend more time in the nests of their host than on their bodies, lowering the likelihood of their collection [77]. Moreover, the fact that mites are more host generalists than fleas [78] may have also contributed to their greater abundance.

The mites in this study were identified as *Laelaps* species, using morphological cues; they did, however, provide the highest-scoring BLAST hit to a sequence from the species *Varroa rindereri* using the cox1 gene. Surprisingly, the mite species was discovered in honeybees in 1998 (accession no: AF107261.2) [3]. *Varroa* was previously known as the “Laelappid-like mite”, and belonged to the family Laelapidea before being separated into the Varroadea family [79]. Thus, the low percent identity value (83.24%) between the DNA sequences suggests that our mites belong to a previously uncharacterized species in the Mesostigmata group. To the best of our knowledge, this is the first study on the molecular identification of rodent mites in Tanzania. Thus, the quite limited sequenced data for Mesostigmata mites and fleas from Tanzania available in GenBank made it difficult to compare our DNA sequence. We only sequenced small samples to confirm the species; thus, further study using different “target genes” is needed to resolve the ambiguity of the mite species identification, and to detail the molecular characteristics of the ectoparasites. Finally, it might also be worth questioning: "Can the same mite species parasitize rodents and honeybees?" 

While there remains much to be learned, our study provided an overview of the key factors that determine the distribution of ectoparasites on the two rodent species; host species, sex, and temperature are predictive variables. Host species influenced parasite prevalence and ectoparasite abundance. This is due to the fact that different species have different resistances (i.e., the capacity of the host to reduce parasite establishment) and tolerance (i.e., the competence of the host to resist a given parasite load and sustain fitness while under infestation) [80]. Moreover, the infestation of the animal may be determined by different factors, such as the color and type of the host’s fur. The greater prevalence and abundance of ectoparasites on *M. delectorum* than on *R. dilectus* may partly be associated with its soft fur, which potentially allows ectoparasites to penetrate easily. However, the greater ectoparasite species diversity (four species) in *R. dilectus* could be due to the striped, white-grey color of the rodent, which could be easily detectable by the ectoparasites, as some species are known to use the host’s color to find their host. Though it may depend on the host–parasite taxon, this can support previous findings that found plumage color affected the infestation of lice on *Columba livia* [81]. Moreover, our study showed that sex was among the predictors of ectoparasite load, with the burden of parasites biased towards male individuals. Similar findings were reported in previous studies on different taxa: rodents [82,83], geckos (*Quedenfeldtia trachyblepharus*) [84], and grey squirrel species [85]. Different factors may contribute to these sex-based variations. Larger male hosts in mammal species [86] provide a wider range of niches for parasites, and can thus support a greater number of parasites [87,88]. In addition, male hosts often have higher energetic requirements, necessitating longer distances travelled in the quest of food, increasing their chances of encountering ectoparasites [89]. Furthermore, due to the physiological differences between males and females, the immunosuppressive properties of testosterone tend to diminish the body’s immunity, causing a substantial decline in male immune fitness [90].

We expected that parasite infestation would be affected by the density of the host; this is due to the idea that hosts in high densities should contain more parasite species than hosts in low densities because of the increased contact between individuals [42]. However, we did not see the influence of host density in this study, and it was not a predictor variable in the parasite distribution, which was in line with the findings of Singleton [91], which reported no association between host density and parasite infestation rate in *Mus musculus*. Thus, other abiotic factors such as temperature, humidity, host burrow structures, and soil type may determine the distribution of the parasite [92].

It is commonly reported that elevation determines organisms’ distribution. However, by itself, elevation above sea level means nothing to a species [93]. The correlated environmental variables that change rapidly over short distances generate and maintain the patterns of abundance and distribution [37,38,93]. Generally, the most obvious change predictor with increasing elevation is linear decrease in temperature [94]. Our study showed that temperature significantly influenced the occurrence of ectoparasites, supporting the idea that temperature is considered to be the most important factor changing elevational distribution of species [24,93]. The lower ectoparasite prevalence and mean abundance at 3500 m, in the site where a daily minimum temperature was 2.7–4.6 °C, implies that the cold conditions of the highlands impose thermoregulatory constraints on ectoparasites, and have a direct effect on their physiology and survival rate. This justifies the fact that as temperature is inversely linked to elevation; warmer temperatures at lower elevations provide more favorable conditions for parasite development than at higher elevations. Another reason for the lower infestation at higher elevations would be due to the cold weather; there is less movement of the hosts, hence reduced contact with other individuals and a lower probability of being infested. Though our study is not species-specific, the findings do corroborate prior studies of parasitic mites on lizards [95], feather mites on birds [44], and fleas on rodents [45].

Disease ecologists and conservation biologists argue that parasites are increasing in abundance through time [31]. Parasite outbreaks are predicted to be a result of habitat change, biodiversity loss, and rising temperatures related to climate change [31,32,33]. Previous studies provided evidence that Mount Meru has shown an increase in mean annual temperature, particularly in the last decade where a 0.37 °C rise in mean annual temperature was recorded [39]. This may provide a platform for parasites to increase in abundance through time. However, for the vast majority of wildlife parasite species, the hypothesis that parasites are increasing in abundance over time remains entirely untested [31]. Our study may provide historical data as a baseline against which to compare contemporary parasite burdens over time.

Some of the ectoparasites and rodents reported in this study have been identified as having medical and veterinary importance [49]. The fleas, *X. cheopis*, *C. calceatus*, and *Dinopsyllus spp.*, for instance, are confirmed efficient vectors of bubonic plague in Tanzania, and have been recovered from rodents involved in plague outbreaks [49]. Bubonic plague is a rodent-borne infectious disease caused by the bacterium *Yersinia pestis*. It affects both humans and animals, and is primarily transmitted by flea bites that jump from rodents to humans [9]. In addition, *C. calceatus* has also been reported as a transmitter of bartonellosis in Rwanda [96]. Bartonellosis is another rodent-borne disease caused by Gram-negative bacteria in the genus *Bartonella*. It affects both humans and wild and domestic animals, and is spread to humans by fleas or contact with flea-infested animals [97]. Moreover, some mites, such as *Laelaps* spp., are vectors for zoonoses such as Q fever and rickettsialpox [98], and Chigger mites for scrub typhus [99]. Moreover, the mite, *Varroa destructor*, is a vector of the deformed wing virus (DWV), a bee-pathogenic RNA virus that causes honeybee (*Apis mellifera)* colony losses worldwide [6]. Therefore, more research on zoonotic pathogens is needed to ascertain the risk of zoonosis. Mount Meru is the centerpiece of the Arusha National Park, and is a popular ecotourism destination. It is home to diverse endemic and threatened species. Mount Meru attracts a large number of tourists and researchers, due to its rich biodiversity and numerous trails for nature enthusiasts to explore. On the other hand, animals in the park interact with these parties, who in turn are likely to interact with the local community. Moreover, wildlife, including rodents, cross the park boundaries and enter human dwellings, coming into direct or indirect contact with people and livestock along the edge of the park. All of these interactions increase the likelihood of anthroponotic and zoonotic pathogen transmission, altering the threat of disease to both animals and humans [49]. As human–wildlife interactions increase, the importance of surveillance for zoonotic diseases cannot be overstated. Given the 65% increase in zoonotic outbreaks in Africa over the last decade [50], recognizing the patterns of parasite distribution among wildlife hosts is of major importance. Our study contributes to a better understanding of the ecology of host–ectoparasite relationships along elevational gradients; this is important in this region, not only for identifying potential ectoparasite vectors, but also for designing and implementing vector-borne disease management programs for wildlife conservation and human health. Moreover, no information is available regarding the role of arthropod ectoparasites in the transmission of zoonotic infectious agents. This study may lay the groundwork for screening rodent hosts and their ectoparasites for potential zoonotic pathogens on Mount Meru. 

## 5. Conclusions

The elevational distribution of ectoparasites infesting *Montemys delectorum* and *Rhabdomys dilectus* was influenced by the host traits (species and sex), in combination with environmental factors such as temperature. The host sex and species influenced the prevalence and abundances of ectoparasites, such that it was higher for males than females and for *M. delectorum* than *R. dilectus*. Our findings did not support our hypothesis that host density predicts the prevalence and abundances of ectoparasites in the two rodent species, but rather that temperature was the best predictor. Since temperatures decrease with elevation, parasite prevalence and mean abundance were lower at higher elevations, highlighting the idea that cold conditions at higher elevations limit reproduction and development—this shows higher elevation zones are ideal for conservation. This may have an impact in light of climate change, since organisms shift their distribution towards higher altitudes. This could also affect species that act as parasite reservoirs and vectors, altering wildlife and livestock infestation patterns. Furthermore, the rodents and ectoparasite species described in this study have been reported to be vectors of diseases of medical and veterinary importance, necessitating precautions. Moreover, Mount Meru is a refuge for a number of endemic and threatened species on the IUCN Red List. Thus, parasitic infection can also be an additional risk to these critical species and biodiversity in general. Therefore, our study lays the groundwork for future wildlife disease surveillance and biodiversity conservation management plans. In addition, the flea and mite species identification using cytochrome oxidase genes in this study at least partly helps to fill the scarcity of sequence data for ectoparasites of African rodents in GenBank. We found a previously uncharacterized mite species in the Mesostigmata group that was previously known to be a parasite of honeybees. Further investigations may shed light into the role of this mite species on Mount Meru.

## Figures and Tables

**Table 1:** Flea and mite species infecting *M. delectorum* and *R. dilectus* at different elevation zones on Mount Meru. N and n are the numbers of hosts examined and infested, respectively. Count and mean abundance of the fleas and mites are provided in brackets.

Host (Infested /Examined)	Ectoparasite Taxa	1500 m N = 6 n = 3	2000 m N = 83 n = 57	2500 m N = 201 n = 130	2950 m N = 70 n = 29	3500 m N = 38 n = 15	Total N = 398 n = 234
*Montemys delectorum* (211/335)	*Xenopsylla cheopis*	0	0	0	0	0	0
	*Dinopsyllus ellobius*	0	22(0.28)	20(0.21)	2(0.05)	0	44
	*Ctenophthalmus calceatus cabirus*	0	0	0	0	0	0
	Mite	0	52(0.67)	121(0.62)	24(0.56)	11(0.60)	208
	Total	0	74(0.95)	141(0.72)	26(0.64)	11(0.60)	252
*Rhabdomys dilectus* (23/63)	*Xenopsylla cheopis*	2(0.05)	0	0	0	0	2
	*Dinopsyllus ellobius*	0	0	2(0.40)	5(0.19)	0	7
	*Ctenophthalmus calceatus cabirus*	0	0	1(0.20)	4(0.15)	1(0.05)	6
	Mite	3(0.75)	0	0	37(1.37)	18 (0.82)	58
	Total	5(1.25)	0	3(0.6)	46(1.70)	19(0.86)	73
	Overall ectoparasites (MA ± SE)	0.16 ± 0.10	0.29 ± 0.17	0.19 ± 0.12	0.16 ± 0.08	0.12 ± 0.09	

MA = mean abundance.

**Table 2:** Flea and mite infestation probability (%P) and ecological dominance index (%D) on *M. delectorum* and *R. dilectus* rodents.

Ectoparasite Taxa	%P	%D
*Xenopsylla cheopis*	0.25	0.62
*Dinopsyllus ellobius*	7.11	15.69
*Ctenophthalmus calceatus cabirus*	0.76	1.85
Over all fleas	8.12	18.15
Mites	50.68	81.85

**Table 3:** Summary of best model describing the ectoparasite abundance (zero-inflated negative binomial) and prevalence (binomial and logitlink function) of the rodents *M. delectorum* and *R. dilectus* on Mount Meru, Tanzania. For categorical variables, the reference level is reported in parentheses.

Variables	Estimate	SE	95% LCL	95% UCL
**Prevalence**				
Host species (Md)	1.22	0.30	0.32	5.21
Host sex (Male)	2.56	0.28	0.39	4.69
Temperature (°C)	0.50	0.12	0.28	3.44
**Abundance**				
Host species (Md)	0.42	0.14	0.04	8.06
Host sex (Male)	0.93	0.12	0.13	9.17
Temperature (°C)	2.10	0.99	1.16	4.48

Md = *M. delectorum;* SE = standard error; LCL = lower confidence interval, UCL = upper confidence interval.

## Data Availability

All of the data for the study are provided in the paper and Supplementary Materials.

## Supplementary Materials

The following supporting information can be downloaded at: https://www.mdpi.com/article/10.3390/biology12030394/s1, Table S1: AICc models for ectoparasite prevalence; Table S2: AICc models for abundance of ectoparasites.
