# Supplementary material for: Arthropod Ectoparasites of Two Rodent Species Occurring in Varied Elevations on Tanzania’s Second Highest Mountain
Source: Biology (Basel). 2023 Mar 2;12(3):394. doi: 10.3390/biology12030394 (PMC10045264; doi:10.3390/biology12030394)
Supplement: Supplementary file 1 [file biology-12-00394-s001.zip › biology-2170461-supplementary.pdf]

Table S1. Binomial and logit-link function model selection results for prevalence of ectoparasites of rodents on Mount Meru. Variables are host species, host sex, host density, elevation and temperature. AICc, Akaike's information criterion corrected for small sample sizes. The smallest ΔAICc (i.e., the difference between the best and other models) is the best fitting model. Best fitting model is shown in **bold.**

S.no	Model	AICc	ΔAICc
**1**	**Prevalence ~ Host species + Host sex + Temperature**	**377.04**	**0**
2	Prevalence ~ Host species + Host sex + Host density+Temperature	382.65	5.61
3	Prevalence ~ Host density + Host sex	382.97	5.93
4	Prevalence ~ Host sex + Host density + Temperature	387.1	10.06
5	Prevalence ~ Host species + Host sex	397.66	20.62
6	Prevalence ~ Host species + Host sex + Host density	399.05	22.01
7	Prevalence ~ Host species + Host sex + Host density + Elevation	412.55	35.51
8	Prevalence ~ Host sex	422.87	45.83
9	Prevalence ~ Elevation + Host species + Host density	514.22	137.18
10	Prevalence ~ Host species	516.19	139.15
11	Prevalence ~ Host density + Host species	516.74	139.7
12	Prevalence ~ Temperature	517.29	140.25
13	Prevalence ~ Host density + Temperature	522.96	145.92
14	Prevalence ~ Elevation	524.07	147.03
15	Prevalence ~Host density	525.82	148.78

Table S2. Zero-inflated model selection results for the ectoparasite abundance of rodents in the Mount Meru. Variables are host species, host sex, host density, elevation and temperature. AICc, Akaike's information criterion corrected for small sample sizes. The smallest ΔAICc (i.e., the difference between the best and other models) is the best fitting model. Best fitting model is shown in **bold.**

	Model	AICc	ΔAICc
**1**	**Abundance ~ Host species + Host sex + Temperature**	**814.90**	**0**
2	Abundance ~ Host species +Host sex	816.95	2.05
3	Abundance ~ Host species +Host sex + Host density	819.99	5.09
4	Abundance ~ Host species +Host sex + Host density + Temperature	824.61	9.71
5	Abundance ~ Host sex + Host density + Temperature	827.44	12.54
6	Abundance ~ Host density +Host sex	829.49	14.59
7	Abundance ~ Host sex	831.68	16.78
8	Abundance ~ Host species	889.61	74.71
9	Abundance ~ Host density + Host species	892.79	77.89
10	Abundance ~ Temperature	893.90	79
11	Abundance ~ Host species + Host sex + Host density + Elevation	895.97	81.07
12	Abundance ~ Host density + Temperature	896.70	81.8
13	Abundance ~ Elevation + Host species + Host density	897.15	82.25
14	Abundance ~Host density	898.29	83.39
15	Abundance ~ Elevation	899.61	84.71
